# Research Progress in the Development of Vaccines against Mycoplasma *gallisepticum* and Mycoplasma *synoviae*

**DOI:** 10.3390/microorganisms12081699

**Published:** 2024-08-17

**Authors:** Shaopeng Wu, Miaoli Wang, Xiaoxue Yang, Lu Zhao, Zouran Lan, Shuhong Sun

**Affiliations:** 1 Shandong Provincial Key Laboratory of Animal Biotechnology and Disease Control and Prevention, College of Animal Science and Veterinary Medicine, Shandong Agricultural University, Tai’an 271018, China; wsp@sdau.edu.cn; 2 Shandong Provincial Center for Animal Disease Control, Jinan 250010, China; miaoliwangchina@126.com (M.W.); 18561896069@126.com (X.Y.); zl19931206@126.com (L.Z.)

**Keywords:** Mycoplasma *gallisepticum*, Mycoplasma *synoviae*, vaccine, attenuated vaccines, genetic engineering vaccine

## Abstract

Mycoplasma *gallisepticum* (MG) and Mycoplasma *synoviae* (MS) are the primary agents responsible for mycoplasma disease in poultry. MG has been identified as a significant cause of chronic respiratory disease in chickens, while MS has been linked to the development of tenosynovitis, joint swelling and other symptoms in chickens, leading to considerable economic losses for the poultry industry. Unfortunately, there is no specific drug for treatment and vaccination is the most important way to control the disease. There are some different types of vaccines, including live vaccines, inactivated vaccines, sub-unit vaccines and vector vaccines. This paper provides a comprehensive review of the development of vaccines for MG and MS.

## 1. Introduction

The primary aetiological agents of avian mycoplasmosis are MG and MS [1]. They have been shown to exhibit hemagglutination activity in the red blood cells of turkeys and chickens [2]. MG can cause chronic respiratory diseases, including rales, coughing, sneezing, runny nose and swelling of the suborbital sinus. It also easily combines with other pathogens, such as avian influenza (AI), Newcastle disease (ND), infectious bursal disease (IBV) and *Escherichia coli*, leading to systemic synovitis and increased mortality [3]. Once parasitized in chickens, MS is susceptible to tenosynovitis, joint swelling and other symptoms. Secondly, the bacteria will produce a large number of toxic substances in chickens, such as neurotoxins, cytolytic enzymes, catalase, etc. These substances affect the cellular function of chickens and are a significant contributing factor to disease development [4]. It can be stated that both MG and MS infection will ultimately result in an increased incidence of carcass condemnation, accompanied by a decrease in egg production, hatchability, feed efficiency and body weight [5].

Chickens must be subjected to regular inspection and vaccination to achieve the desired level of purification. At present, vaccination represents the primary method of preventing the occurrence of MG and MS. MG and MS vaccines can be broadly categorized into three main groups: live attenuated vaccines, inactivated vaccines and genetically engineered vaccines. In the context of next-generation vaccine development, further evaluation of the screening methods and identified antigens from the Protein Modules & Combinatorial Peptide Library will be instrumental in the development of new vaccines that will more effectively prevent MG and MS. The poultry industry has suffered significant economic losses due to the high pathogenicity of these pathogens. This paper provides a comprehensive review of the development status of various vaccines. The objective is to establish a theoretical foundation for the creation of novel vaccines that can provide augmented protection in regions impacted by MG and MS.

## 2. Conventional Vaccines

### 2.1. Inactivated Vaccine

Inactivated vaccines have been employed for the control of mycoplasma disease in poultry since the 1960s [6]. Due to the increase in antibiotic resistance, the efficacy of antibiotics in controlling MG infection is decreasing; as such, it needs to be combatted with new and effective vaccines that can be created through the enhancement of existing vaccines. MG generally needs to be cultivated in mycoplasma culture media to an appropriate concentration and then concentrated for vaccine production. Many MG isolates have been used to produce inactivated vaccines across a range of countries and regions. It has been demonstrated that the R strain can reduce respiratory symptoms, respiratory disease, egg transmission and the production losses associated with MG [7]. In addition to univalent inactivated vaccines, multivalent inactivated vaccines are also used to prevent MG infection. For example, pentavalent inactivated vaccines for chickens against five pathogens, namely Salmonella typhimurium, Salmonella enteritidis, Salmonella kentucky, MG and MS, have demonstrated favorable preventive effects against salmonellosis and mycoplasma infections [8]. At present, the inactivated MS vaccine has not been commercialized. Gong et al. investigated the protective effects of ISA 71 VG and chitosan as adjuvants of the inactivated MS vaccine. The findings indicated that the inactivated MS vaccine containing ISA 71 VG could induce both cellular and humoral immune responses in broilers, thereby conferring a high level of protection [9]. Compared with the live attenuated vaccine, the oil emulsion inactivated vaccine offers the greatest advantages in terms of reducing virulence and inducing high levels of humoral antibodies [10], which is convenient for storage. However, the disadvantage is that it requires multiple immunizations to boost the immune system, resulting in higher costs.

### 2.2. Live Attenuated Vaccines

Currently, three distinct strains of the commercially available live attenuated MG vaccine are available in China: the F strain [11], the ts-11 strain [12] and the 6/85 strain [13]. Previously published articles have provided a comprehensive overview of commercially available MG vaccines [14]. It has been demonstrated that all of these vaccines are capable of providing effective protection in chickens when used in commercial flocks, but the three live vaccines exhibited differences in terms of their protective efficacy, pathogenicity and transmission. In comparison to the F strain, the ts-11 strain and the 6/85 strain have been demonstrated to be safer [15]. A previous report evaluated the efficacy of MG vaccines in a co-infection model and suggested that ts-11 and 6/85 provided some protection against the virulent MG strain. The two kinds of vaccines provided non-specific protection. However, ts-11 was more effective than 6/85 in the trachea, bursa and air sacs, but not in the lungs [16]. As a live vaccine candidate, strain K (K5831) has an excellent safety profile and demonstrates the same protective efficacy as the F strain and the ts-11 strain, indicating its significant potential for application [17]. The mutant of the ts-11 strain (ts-304) can also be used as a candidate live vaccine and a 10-fold increase in the inoculation dose is also safe, rendering it a suitable and efficacious candidate live vaccine for turkeys [18]. The use of attenuated vaccines has been demonstrated to result in a more robust protective effect and a longer duration of protection. However, a study from Egypt reported the spread of the live F strain from vaccinated chickens to unvaccinated chickens [19]. Moreover, there is a potential risk of the ts-11 strain becoming stronger and potentially spreading, which may impact its suitability for use [20]. Core genome MultiLocus Sequence Typing (CgMLST) showed that ts-11-like isolates obtained from commercial broiler outbreaks in northeastern Georgia were closely related to ts-11 vaccine strains [21]. With the development of technology, many candidate vaccines for MG, such as GT5 [22], MG 7 [23], K strain, and ts-304 [18], have been investigated as potential replacements to address concerns regarding the efficacy and safety of existing vaccine strains. The candidate vaccines GT5 and MG 7 are derived from the virulent strain Rlow. The continuous passage of the virulent Rlow strain has led to the emergence of the Rhigh strain, which was then complemented with the cell adhesion gene *GapA* to create the GT5 vaccine strain [24]. The MG 7 strain was developed by inserting a transposon in the middle of the virulent dihydrothionylamide dehydrogenase gene [25]. Recently, Condello et al. reported that a novel, attenuated vaccine, Vaxsafe MG304, administered via eye-drops to a one-day-old chicken [18] demonstrated a protective immunity equivalent to immunization at 3 weeks of age [26].

Only two live attenuated vaccines for MS are currently available on the commercial market: the temperature-sensitive (ts) MS-H vaccine strain (Vaxsafe MS, Bioproperties Pty Ltd., Ringwood, VIC, Australia) and the NAD-independent MS1 vaccine strain (Nobilis MS Live, MSD Animal Health Inc., Rahway, NJ, USA). The MS-H strain was developed through a chemical mutation of the Australian strain (86079/7NS) and became a commercial vaccine. It was temperature-sensitive and registered in China in 2017 [27]. There are two principal methods of inoculation: eye-drops and a spray. Through a controlled experiment, the clinical manifestations of vaccinated chickens infected with the disease were found to be milder than those of unvaccinated chickens, and the incidence rate was relatively low. In addition, the MS-H strain has been demonstrated to exert a marked effect in the reduction of eggshell abnormalities resulting from MS infection [28]. Notably, when chickens are infected, the administration of a vaccine will not only prove ineffective in providing protection but may even aggravate the condition.

## 3. Genetic Engineering Vaccine

Genetic engineering vaccines encompass a multitude of varieties, predominantly comprising vaccines prepared through genetic engineering technology. These vaccines exhibit disparate preparation methodologies and application characteristics. The principal categories of genetic engineering vaccines are as follows: genetic engineering subunit vaccine, vector vaccine, DNA vaccine, synthetic peptide vaccine, gene deletion vaccine, and transgenic plant vaccine. While genetic engineering vaccines offer the benefits of improved safety and low cost, they require sophisticated technology and equipment.

### 3.1. Subunit Vaccine

Recently, there has been a notable increase in interest surrounding recombination vaccines. The vaccine comprises the identification and cloning of immunogenic molecules in an appropriate expression system [29]. Functional transposons in mycoplasma (such as Tn916 and Tn4001) have been employed for the construction of mutants, the expression of genes, the analysis of cell markers and the investigation of protein function [30]. Two recombinant candidate vaccines against MG infection have been developed: GT5 and a fowlpox virus encoding the MG gene [22,29]. In a recent study, Zhang et al. used a recombinant adenovirus to express the S1 spike glycoprotein of IBV and the TM-1 protein of MG in HEK293 cells [31]. The recombinant adenovirus retained the biological characteristics of the parents, successfully expressed the target protein, produced high-level antibodies and significantly reduced the clinical signs and lesions after IBV and MG attacks [32]. It was reported that a significant protective effect on MS infection was observed when DNAK, enolase, elongation factor Tu (EF-TU), immunodominant and surface-exposed membrane proteins B (MSPB), NADH oxidase and lipoprotein 78 (LP78) proteins were expressed [33]. The number of DNA copies in the trachea was significantly reduced, the score of the air sac lesion was low and the thickness of the tracheal mucosa was minimal. Unfortunately, this approach is only capable of reducing lesions caused by MS infection, rather than acting as a means of preventing MS [33]. Furthermore, the number of synovial mycoplasma antigens was limited, including enolase [34], the pyruvate dehydrogenase complex E1 α and β subunits (PDHA and PDHB), dihydrothionyl amide dehydrogenase (PdhD) [35], P35 [36] and NADH oxidase [37]. Zhang employed immunoprotein omics and reverse vaccinology to analyse RS01790 (putative carbohydrate ABC transporter lipoprotein), BMP (substrate binding protein of the BMP family ABC transporter), GrpE (nucleotide exchange factor), RS00900 (putative nuclease) and RS00275 (uncharacterized protein) and found that they exhibited good immunogenicity [38]. The identification of B cell and T cell epitopes is of great significance in the development of vaccines based on multiple epitopes. B-cell epitopes play a pivotal role in the initiation of the humoral immune response [39], whereas T-cell epitopes that are recognized by T-cells can induce a cellular immune response and enhance overall immune activation [40]. The advancement of computational immunology and vaccine informatics has led to the emergence of a plethora of sophisticated tools that facilitate the precise design and development of epitope-based vaccines [41]. Furthermore, the B-cell epitope, MHC-I and MHC-II epitopes of the aforementioned five proteins are connected by appropriate linkers to synthesize a multi-epitope vaccine, which has been demonstrated to have a favorable protective effect [42]. Mugunthan et al. devised a multi-epitope vaccine comprising cytotoxic T-cells, helper T-cells and B-cell epitopes of antigen protein through immunoinformatics, which exhibits the benefits of immune specificity, compactness, and the absence of adverse reactions [43]. The combination of multi-epitope vaccines with immunogenic adjuvants has the potential to elicit a robust immunogenic response, addressing the limitations of existing vaccines [44].

The utilization of plant vaccines presents a multitude of potential advantages, including the capacity to achieve high yields, rapid manufacturing, enhanced safety, and cost-effectiveness [45]. Furthermore, it is improbable that these vaccines will be contaminated by unusual mammalian pathogens. Mugunthan devised a 21.4 kDa multi-epitope peptide vaccine utilizing the immunogenic fragment of cell adhesion protein and expressed it in tobacco leaves. In comparison to the control group, the treatment group exhibited a markedly elevated production of immunoglobulin Y (IgY) neutralizing antibodies against cell adhesion protein epitopes in immunized chickens [46]. Furthermore, it was observed that the oral administration of ST1814G-MG provided superior protection against MG infection in comparison to the inactivated vaccine. Additionally, the co-administration of the two vaccines exhibited augmented efficacy [47].

### 3.2. Recombinant Vector Live Vaccine

The Vectormune FP-MG vaccine is a recombinant fowlpox virus that expresses the 40 k and *mgc* genes of MG. It has been demonstrated to be highly safe. It has been demonstrated to be effective in preventing infection by the fowlpox virus and MG, and has obtained commercial licensing in the United States [29]. Ts-11 was developed as a transposon-based delivery vector to express and secrete chicken IFN-γ (ts-11 C3), which enhances host cell immunity, but not humoral immunity, and may also stimulate the infiltration of mucosal heterophiles [48].

### 3.3. Gene Deletion Vaccine

Inoculation with the oppD1 mutant resulted in the complete protection of the air sac and trachea from damage caused by the pathogenic MG. Furthermore, the infection’s impact on weight gain is reduced, and the toxic MG is partially prevented from establishing itself in the trachea [49]. As a recombinant, attenuated, live vaccine, the cell adhesion gene deletion strain CT5 is capable of eliminating the pathogen by reducing the adhesion and colonization of MG in cells, thereby conferring a robust protective effect on chickens [50].

### 3.4. Immune Program and Immunologic Adjuvant

The protective efficacy of different vaccine strains varies, as does the protective efficacy of the same vaccine strain when administered using different vaccination procedures [51]. The administration of vaccines via eye drops can enhance the efficacy of the vaccination process [52]. An adjuvant is defined as an immune-stimulatory component that enhances the magnitude and durability of immune responses induced by vaccination, even with low doses of antigens [53]. In general, aluminium hydroxide is the most commonly used adjuvant system and has been demonstrated to induce a robust humoral immune response [54]. It has been demonstrated that chitosan can be employed as an efficacious mucosal adjuvant, thereby enhancing the potency of MG vaccines [55,56]. Moreover, whether a combination of toll-like receptor (TLR)1/2 and TLR3 agonists (L-pampo) can be a potent adjuvant for severe acute respiratory syndrome coronavirus 2 subunit vaccine [54]. In recent years, a substantial body of evidence has emerged confirming the efficacy of IL-2 and IL-6 as ideal cytokine immune enhancers. Furthermore, these genes have been successfully cloned and expressed in animal models, providing a promising foundation for further research. Therefore, the trend in the development of a new generation of DNA vaccines or gene immunization will be to construct a plasmid that co-expresses IL-2 with other antigen genes. This method goes further than the method of mixing IL-2 with vaccine or mixing IL-2 gene expression plasmid with target antigen gene vaccine. The recombinant protein obtained in this way can not only improve the vaccine effect but also enhance the immunity of animals as a whole. Therefore, combining some protein genes of MG with chicken IL-2 or IL-6 genes for gene immunization may provide a new way to prevent MG infection.

## 4. Prospects and Summary

The list of currently available vaccines against MG and MS infection is given in Table S1. It is very important to develop an effective vaccine against MG and MS. It would also be highly beneficial if a single vaccine could prevent and control two diseases at the same time. At present, most vaccine development strategies are based on a single antigen or different antigens. However, the single-injection approach remains the prevailing method. Therefore, the novel vaccine based on an epitope is considered to be an excellent futuristic method. Antigens contain epitopes, which are the fundamental units capable of eliciting cellular or humoral immune responses. A multi-epitope vaccine consists of a series of epitope (antigen) peptides, which is helpful in preventing infection or inducing an immune response.

The majority of vaccine expression systems currently employed for the production of multi-epitope vaccines are based on bacteria, yeast and mammals. However, these traditional vaccine production systems are associated with a number of disadvantages. For instance, the bacterial expression system is prone to difficulties in the expression of natural structural proteins, endotoxin accumulation, and host protease pollution. In the yeast expression system, the main disadvantage is high glycosylation, whereas the major drawbacks of the mammalian expression system are the high cost, slow cell growth and elevated pollution potential.

Some candidate immune proteins of the MG vaccine have been identified, such as GroEL, EF-Tu, greA, PDHC and Dnak, and it has been found using proteomics that they have a good immunogenicity [57]. Further research on the GroEL protein found that it has ATPase activity and participates in the refolding of te MG PrpC protein. At the same time, the rabbit antiserum of GroEL has a good bactericidal effect, which is similar to the antiserum induced by an inactivated vaccine, suggesting that GroEL is a protective antigen and can be used as a candidate antigen for the MG subunit vaccine [58]. MGC1 and MGC2 are important adhesion factors of MG. The subunit vaccine made of MGC1 and MGC2 heavy histone mixed with oil adjuvant has a strong immune response, which proves that MGC1 and MGC2 heavy histone have a good immunogenicity and can induce antibody production. Bercic et al. performed two-dimensional electrophoresis combined with a Western blot test on the MG ULB 02/P4 and ULB 02/OV6 strains. This approach enabled the identification of several immune-related proteins, including PdhD, elongation factor EF-G, pyruvate kinase, NADH oxidase, ATP synthase, trigger factors, DnaK, P70, P110, P160, LP85 and LP78 [59].

Multi-epitope vaccines against the following poultry pathogens have been developed: Newcastle disease virus, avian influenza A (H7N9) virus [60], Emile parasite infection [61] and Herpesvirus of Turkey (HVT) vector vaccine. As a widely used vaccine vector, HVT has been applied to control several avian diseases, including AI [62], IBD [63] and NDV [64] by encoding heterologous antigen proteins as dual or triple vaccines [65]. High-quality antigen delivery systems have been put into use one after another, such as live attenuated typhoid vaccine [66] and adenovirus [67], with more effective adjuvants such as IMS 1113 [68], lithium chloride [69], mesoporous silica nanoparticles [70] and so on to enhance the immune protection effect of vaccines, providing the scientific basis for the development of novel vaccines.

The use of vaccines is the main means of preventing and controlling MG and MS, especially in commercial laying hens and breeder flocks. However, in some cases, even with vaccination, infection can occur suddenly. For example, latent infection and transmission between different flocks and within the same flock by horizontal and vertical routes are possible. Therefore, it is recommended that biological surveillance and biosecurity measures are implemented to completely eradicate infected chickens.

## Data Availability

The original contributions presented in the study are included in the article; further inquiries can be directed to the corresponding authors.

## Supplementary Materials

The following supporting information can be downloaded at: https://www.mdpi.com/article/10.3390/microorganisms12081699/s1, Table S1: The list of currently available vaccines against MG and MS infection.
