# Supplementary material for: Research Progress in the Development of Vaccines against Mycoplasma gallisepticum and Mycoplasma synoviae
Source: Microorganisms. 2024 Aug 17;12(8):1699. doi: 10.3390/microorganisms12081699 (PMC11356929; doi:10.3390/microorganisms12081699)
Supplement: Supplementary file 1 [file microorganisms-12-01699-s001.zip › microorganisms-3134171-supplementary.pdf]

Table. S1 The list of currently available vaccines against MG and MS infection

Types of vaccines	MG vaccines	MS vaccines
Inactivated vaccine	R strain[7]	-
Live-attenuated vaccines	F strain[11], ts-11 strain[12], 6/85 strain[13], K strain (K5831)[17], ts-304[18], MG 7[23] and Vaxsafe MG304[26].	MS-H vaccine strain[27] and the NAD-independent MS1 vaccine strain
Genetically engineered vaccines	GT5[22], Vectormune FP-MG vaccine[29], ts-11 C3[48] and CT5[50].	-
